# Salivary IL-1ß as an Objective Measure for Fatigue in Multiple Sclerosis?

**DOI:** 10.3389/fneur.2018.00574

**Published:** 2018-07-16

**Authors:** Katrin Hanken, Carina Sander, Lara Qaiser, Hans-Peter Schlake, Andreas Kastrup, Michael Haupts, Paul Eling, Helmut Hildebrandt

**Affiliations:** ^1^Klinikum Bremen-Ost, Department of Neurology, Bremen, Germany; ^2^Department of Psychology, Carl von Ossietzky Universität Oldenburg, Oldenburg, Germany; ^3^Rehabilitationszentrum Oldenburg GmbH, Oldenburg, Germany; ^4^Augustahospital Anholt, Clinic for Neurology, Anholt, Germany; ^5^Department of Neurology, Heinrich-Heine-Uni, Dusseldorf, Germany; ^6^Donders Institute for Brain, Cognition and Behaviour, Radboud University Nijmegen, Nijmegen, Netherlands

**Keywords:** multiple sclerosis, disease course, fatigue, inflammation, proinflammatory cytokines, IL-1ß

## Abstract

**Background:** The causes of fatigue in multiple sclerosis (MS) and other inflammatory disorders are not well understood. One possible cause that might explain fatigue in inflammatory disorders appears to be the immunological process itself, triggering neural activity that is experienced as fatigue.

**Objectives:** To investigate whether salivary IL-1ß concentration, associated with systemic inflammation, is related to subjective fatigue in MS.

**Methods:** 116 MS patients (62 relapsing remitting MS, 54 secondary progressive MS) and 51 healthy controls participated in this study. Salivary concentration of IL-1ß was determined using a commercially available enzyme-linked immunosorbent assay (ELISA) kit. Fatigue was assessed using various fatigue scales. We compared IL-1ß concentration between groups and performed regression analyses to investigate which variables best predict fatigue scores.

**Results:** We found that the IL-1ß concentration best predicts fatigue scores in relapsing remitting MS patients, even though the IL-1ß concentration did not differ significantly between relapsing remitting MS patients and healthy controls. Secondary progressive MS patients showed a somewhat elevated IL-1ß concentration compared to relapsing remitting MS patients and healthy controls. Furthermore, disease modifying treatment had a significant effect on the IL-1ß concentration, with treated patients showing a lower IL-1ß concentration than non-treated patients.

**Conclusions:** The present study points to a significant relation between the proinflammatory cytokine IL-1ß and fatigue in relapsing remitting MS patients. It also suggests a potential effect of disease modifying treatment on the peripheral IL-1ß concentration.

## Introduction

Fatigue arguably presents the most challenging symptom for a majority of multiple sclerosis (MS) patients (1). Its prevalence ranges from 65 to 97% and it tends to seriously impair approximately one-third of all MS patients (1–3). Apart from having negative effects on a patient's social and private life, fatigue imposes significant socioeconomic consequences and is a major reason for the reduction of working hours and early retirement (4–6). Despite this serious negative impact on daily life activities, fatigue is still poorly understood and often under-estimated. That is why we recently developed a model explaining the origin and consequences of MS-related fatigue (7). According to our model, subjective fatigue in MS patients is related to peripheral inflammation. The feeling of fatigue in MS patients is considered a form of sickness behavior, resulting from cytokine-mediated activity changes within brain areas involved in interoception such as the hypothalamus, the amygdala, the insula and the anterior cingulate. Looking at proinflammatory cytokines, IL-1ß is one of the main mediators of sickness behavior. IL-1ß activates afferent vagal neurons and it has been strongly and consistently linked to symptoms of sickness behavior including fatigue (8–11).

To date, only few studies have investigated the association between subjective fatigue in MS patients and systemic inflammation (12–18). Studies investigating the characteristics of peripheral T lymphocytes frequently found increased production capacities for proinflammatory cytokines such as IFN-γ and TNF-α in fatigued MS patients (14, 15, 17). Other studies reported a higher serum concentration of proinflammatory cytokines (IL-6, IL-1ß) in patients with high levels of self-reported fatigue (13, 16). It has been argued that increased peripheral inflammatory processes cannot explain fatigue in progressive MS patients since progressive MS is characterized by diffuse central nervous system atrophy and new inflammatory lesions are rare in these disease stages (19). In our view, the fact that there are no relapses any more does not necessarily imply that there are no underlying inflammatory processes in the body periphery (19). Hardly any study investigated the relation between proinflammatory cytokines and fatigue in chronic disease stages of MS, or compared the proinflammatory cytokine concentration of MS patients with relapsing-remitting (rr) and secondary progressive (sp) MS. Hence, we wanted to investigate whether there is a difference in the concentration of proinflammatory cytokines between rrMS and spMS patients and whether proinflammatory cytokines may predict fatigue scores in MS patients suffering from different clinical disease courses. Furthermore, few studies indicate that a disease modifying therapy alters cytokine production in MS patients (20–23). Thus, we also aimed to investigate whether disease modifying drugs have an effect on peripheral markers of inflammation.

Most biomarkers that are present in blood and urine can also be detected in a sample of saliva. Several inflammatory markers have been reliably determined from saliva and some studies reported even higher levels of inflammatory markers in saliva than in blood (24–27). Riis et al. (26) detected higher levels of the proinflammatory cytokine IL-1ß in saliva than in blood serum, and they also found a moderate correlation between these two measures in healthy adolescent girls. Hence, saliva, a non-invasive method for measuring salivary concentration of IL-1ß, may be a promising tool for monitoring patients with systemic inflammatory diseases.

Because we assume that systemic inflammation contributes to fatigue in rrMS as well as in spMS patients, we compared the salivary IL-1ß concentration and investigated which variables best predict fatigue for rrMS and spMS patients.

## Materials and methods

### Study population

From August 2015 till the end of June 2017, inpatients of the Klinikum Bremen-Ost, Bremen, Germany, the Augusta Hospital Anholt, Anholt, Germany and patients from the Median Clinic Wilhelmshaven, Wilhelmshaven, Germany were asked to participate in the study. Additionally, MS patients were recruited from MS support groups in Bremen and surroundings. A total of 116 patients with rrMS (*n* = 62) and spMS (*n* = 54) participated in this multi-center study. Pregnant patients or individuals with an MS relapse or using corticosteroids during the last 4 weeks before assessment, under legal care and/or with a diagnosis of any other neurodegenerative disease were excluded from the study. Additionally, 51 healthy controls participated. The study was approved by the ethical board of the German Society of Psychology (DGP) and written informed consent was obtained from participants.

### Clinical investigation

Clinical status of all patients was assessed with the Expanded Disability Status Scale [EDSS; (28)]. Fatigue was assessed with two self-reported questionnaires, the Fatigue Severity Scale [FSS; (29)] and the Fatigue Scale for Motor and Cognitive Functions [FSMC; (30)]. The FSS consists of nine items assessing severity and frequency of fatigue, with higher scores representing stronger fatigue. The FSMC evaluates two main components of fatigue, namely cognitive and motor fatigue. It is composed of 20 items. The cut-off score between normal and mild pathological fatigue is 43 for the total scale and 22 for the cognitive and motor scale.

Depressive mood was investigated using the Beck Depression Inventory Scale [BDI; (31)]. The items A-O, the psychological items, were used to calculate the score for mood impairment whereas items P-U (sleep, tiredness, body weight, loss of sexual interest, somatic concerns) reflect the somatic score and were excluded from further analysis (32, 33).

### Saliva collection

Participants were asked not to drink, eat, brush their teeth, smoke or chew gum at least for 1 h prior to the examination on the day of saliva collection. Whole unstimulated saliva was collected using standard techniques according to Navazesh (34). Participants were asked to swallow first, tilt their head forward and then letting saliva pool in their mouth for 5 min. Each minute, participants were instructed to gently spit their saliva into a sterile 100 ml container. Saliva samples were immediately refrigerated and stored at −20°C.

### Salivary IL-1ß analysis

The analysis of the saliva samples was performed at the Department of Biochemistry of the Carl von Ossietzky University Oldenburg. On the day of the analysis, samples were stored for approx. 30 min at room temperature to defrost. 1000 μl of each sample was pipetted into a tube and centrifuged for 15 min at 1000 × g. IL-1ß levels in salivary supernatants were determined using a commercially available enzyme-linked immunosorbent assay (ELISA) kit (Quantikine® ELISA Human IL-1β/IL-1F2 Immunoassay, R&D Systems, Inc., Minneapolis, MN, USA) according to the manufacturer's instructions for serum/plasma samples. The minimum detectable dose of human IL-1β is typically less than 1 pg/ml. Samples were analyzed in duplicate and the absorbance was measured at 450 nm (wavelength correction was set at 620 nm). The concentration of the samples was calculated from the standard curve. The results are presented in picogram per milliliter (pg/ml).

### Statistical analysis

**Figure 1:**
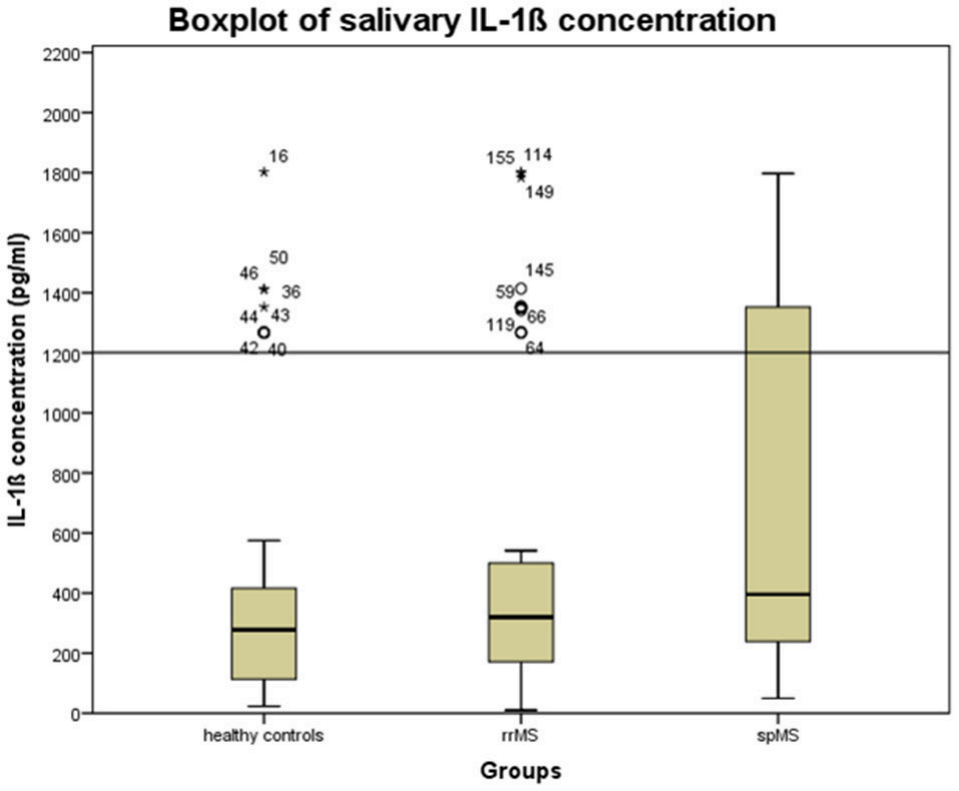
Boxplot of the salivary concentration of IL-1ß in the whole sample to determine the upper cut-off scores for excluding possibly spoiled samples. The error bars reflect the standard deviation. Stars, circles, and numbers display IL-1β concentration of study participants. rrMS, relapsing remitting multiple sclerosis; spMS, secondary progressive multiple sclerosis.

We first checked whether the IL-1ß scores deviated from a normal distribution using the Kolmogorov-Smirnov Test. As they were not normally distributed and since the manufacturer of the ELISA kit recommends laboratory based cut-off values, we performed an outlier analysis on the IL-1ß results to exclude possibly spoiled samples from further analyses. This outlier analysis was based on a boxplot for the healthy control samples and determined the 1.5 ^*^ interquantile score, considering an IL-1ß concentration beyond 1,200 pg/ml as an outlier (see Figure 1).

We subsequently compared IL-1ß concentration between rrMS patients, spMS patients and healthy controls, controlling for age, gender, depressive mood (psychological item score of the BDI) and disease modifying drugs using an univariate analysis of covariance (ANCOVA).

In a final step, we performed separate linear forward regression analyses for the three groups to check which variables best predict the variance in the fatigue scores of rrMS patients, spMS patients and healthy controls. The fatigue scores were defined as dependent variables. IL-1ß concentration, age, gender, number of completed school years, the psychological BDI score and status on disease modifying treatment were defined as independent variables.

## Results

After applying the cut-off score of IL-1ß concentration <1.200 pg/ml, the salivary IL-1ß concentration showed a normal distribution. Applying the cut-off score reduced the groups to 45 rrMS patients (73% of the original sample), 35 spMS patients (65% of the original sample) and 41 healthy controls (80% of the original sample). Table 1 presents the characteristics and mean scores of the groups.

**Table 1:** Group characteristics.

	**rrMS**		**spMS**		**Healthy controls**	
	**Male: 7, female: 38**		**Male: 6, female: 29**		**Male: 10, female: 31**	
	**DMT: 34 (76%)**		**DMT: 11 (31%)**			
	**Mean**	***SD***	**Mean**	***SD***	**Mean**	***SD***
Age (years) ^a^^,^^c^	44.0	10.9	52.1	10.6	41.8	16.2
Time since diagnosis (months) ^a^	111.0	84.8	228.7	103.5		
EDSS ^a^	3.4	2.0	5.8	1.5		
Cognitive fatigue score (FSMC) ^b^^,^^c^	31.8	9.9	33.8	9.7	18.2	7.8
Motor fatigue score (FSMC) ^a^^,^^b^^,^^c^	34.1	9.2	38.3	6.1	18.5	7.5
Total fatigue score (FSMC)^b, c^	65.9	17.2	72.1	14.5	36.7	14.8
Fatigue severity scale score^b, c^	42.0	15.6	41.8	15.9	24.4	11.3
Psychological item score (BDI)^c^	5.2	4.1	6.5	5.2	3.6	4.2
IL-1beta (pg/ml)^c^	242.9	126.6	294.1	168.1	222.7	138.0

a *significant difference (p < 0.05) between rrMS and spMS*.

b *significant difference (p < 0.05) between rrMS and healthy controls*.

c *significant difference (p < 0.05) between spMS and healthy controls*.

*BDI, beck depression inventory; DMT, disease modifying therapy; EDSS, expanded disability status scale; FSMC, fatigue scale for motor and cognition; rrMS, relapsing remitting multiple sclerosis; SD, standard deviation; spMS, secondary progressive multiple sclerosis*.

There was no significant difference in gender distribution between the three groups. As expected, spMS patients were significantly older than rrMS patients and healthy controls, and they had a longer disease duration and a higher EDSS score than rrMS patients. Significantly more rrMS patients received a disease modifying therapy than spMS patients (76 vs. 31%, *p* < 0.001). 70% of MS patients suffered from moderate cognitive fatigue and 83% suffered from moderate motor fatigue. 64% had a FSS score higher or equal than 4 indicating pathological fatigue. RrMS and spMS patients did not differ significantly in the cognitive fatigue score of the FSMC and the FSS score. Only on the motor score of the FSMC, spMS patients scored significantly higher than rrMS patients. 94% of spMS and 76% of rrMS patients suffered from moderate motor fatigue. Overall, rrMS and spMS scored significantly higher on all fatigue scores than healthy controls. RrMS patients and healthy controls did not differ significantly in age. RrMS patients did not differ significantly on the psychological items score of the BDI between spMS and healthy controls. But spMS patients scored significantly higher on the psychological items of the BDI than healthy controls. There was no significant difference in the IL-1ß concentration between rrMS patients and healthy controls or between rrMS and spMS patients. But, spMS patients presented a significantly higher IL-1ß concentration than healthy controls.

**Figure 2:**
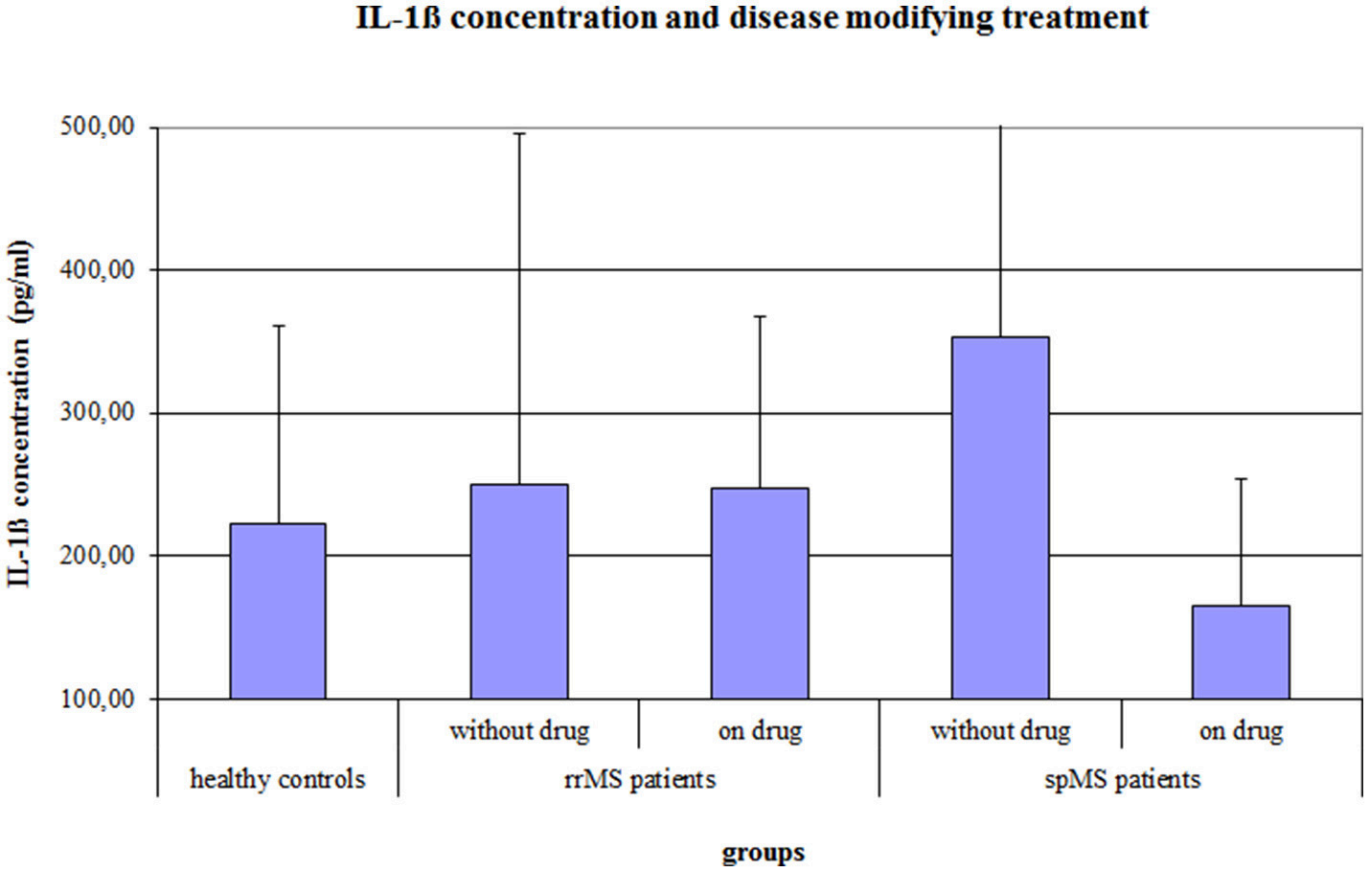
Salivary IL-1ß concentration (pg/ml) of healthy controls and of rrMS and spMS patients with and without a disease modifying therapy. Error bars present the standard deviation. rrMS, relapsing remitting multiple sclerosis; spMS, secondary progressive multiple sclerosis.

The ANCOVA on the IL-1ß concentration, controlling for age, gender, the psychological symptom BDI score and a disease modifying therapy, revealed a significant Group effect (*F* = 4.037, *p* = 0.020) and a significant effect of a disease modifying therapy (*F* = 5.498, *p* = 0.021). In absolute terms, spMS patients showed the highest IL-1ß concentration, but *post-hoc* Bonferroni corrected *t*-tests revealed no significant differences between the groups. Further, MS patients with a disease modifying therapy had a significantly lower IL-1ß concentration than MS patients without disease modifying therapy (222.9 vs. 319.9 pg/ml, *p* = 0.008). The difference in IL-1ß concentration between treated and non-treated patients was larger in the group of spMS patients (164.8 vs. 353.4 pg/ml, *p* = 0.001) than in the group of rrMS patients (241.7 vs. 246.7 pg/ml, *p* = 0.9; see Figure 2).

**Figure 3:**
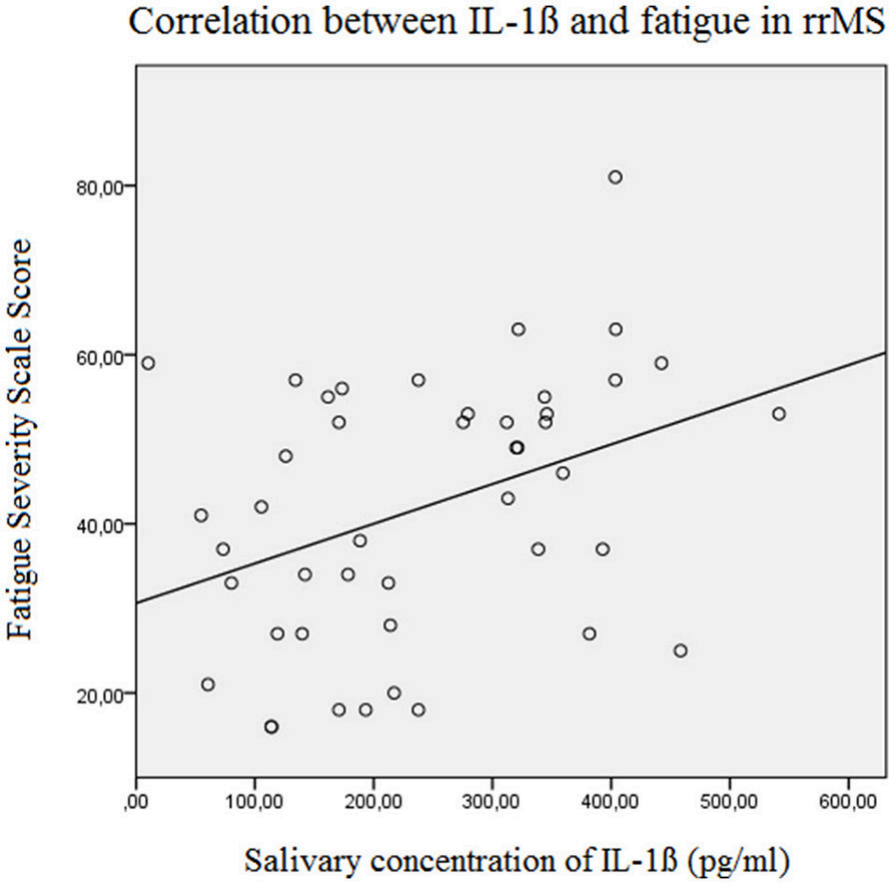
Correlation between the salivary IL-1ß concentration (pg/ml) and the Fatigue Severity Scale Score in relapsing remitting MS patients (Beta = 0.382, *F* = 7.3, *p* = 0.010). rrMS, relapsing remitting multiple sclerosis.

**Figure 4:**
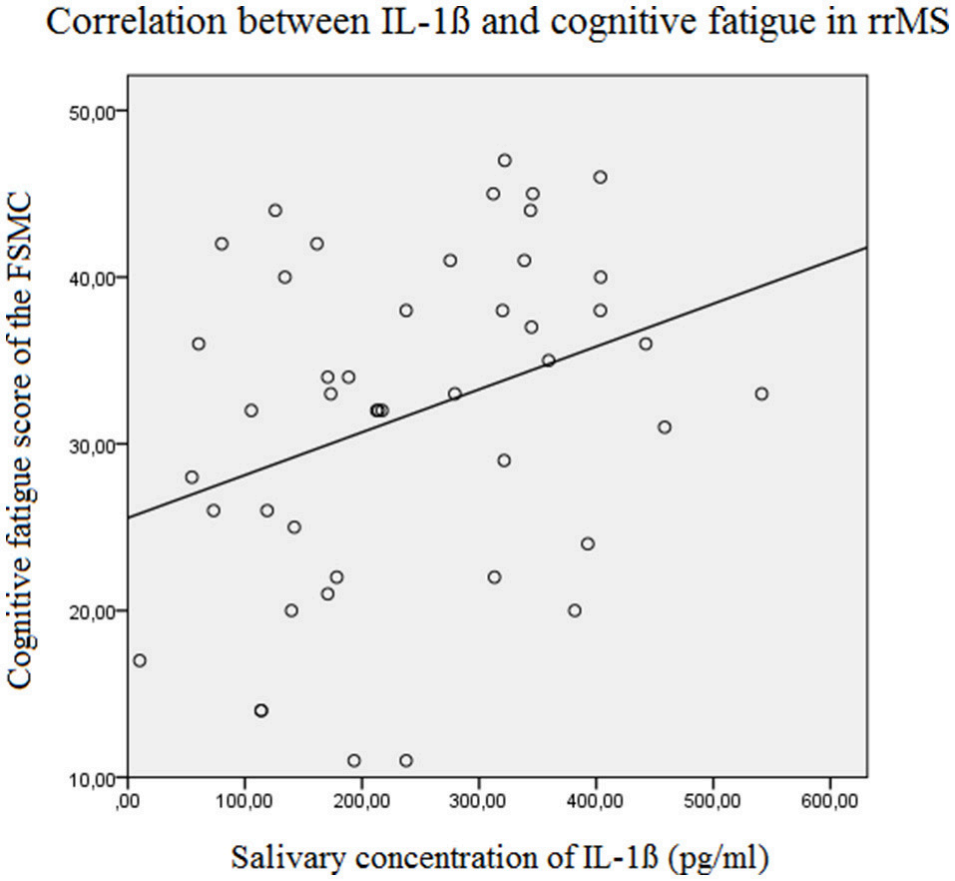
Correlation between the salivary IL-1ß concentration (pg/ml) and the Cognitive Fatigue Score of the Fatigue Scale for Motor and Cognition in relapsing remitting MS patients (Beta = 0.329, *F* = 5.2, *p* = 0.027). FSMC, Fatigue Scale for Motor and Cognition, rrMS, relapsing remitting multiple sclerosis.

The results of the linear forward regression analyses, separate for groups, are presented in Table 2. The results revealed that the cognitive fatigue score of the FSMC of rrMS patients is best predicted by the IL-1ß concentration. The model explains a significant percentage of the variance of the cognitive fatigue score (*R*^2^ = 0.108, *F* = 5.223, *p* = 0.027). The motor fatigue score of the rrMS patients can be best predicted by a model including the psychological BDI score, disease modifying therapy and the concentration of IL-1ß (*R*^2^ = 0.438, *F* = 10.657, *p* < 0.001). The FSS score of rrMS patients can be best predicted by a model including disease modifying therapy, the IL-1ß concentration, the psychological BDI score and a patient's age (*R*^2^ = 0.439, *F* = 7.828, *p* < 0.001). In all models, the IL-1ß concentration positively correlated with the fatigue scores (see Figures 3, 4). For spMS patients, none of the independent variables significantly predicted fatigue scores of the Fatigue Scale for Motor and Cognition. The FFS of spMS patients was best predicted by a model including age (*R*^2^ = 0.147, *F* = 4.294, *p* = 0.049). For healthy controls, the psychological BDI score was included in the models best predicting the different fatigue scores of the FSMC (cognitive fatigue score: *R*^2^ = 0.489, *F* = 37.345, *p* < 0.001; motor fatigue score: *R*^2^ = 0.373, *F* = 23.179, *p* < 0.001). The FSS of healthy controls was best predicted by a model including the psychological BDI score and age (*R*^2^ = 0.251, *F* = 6.365, *p* = 0.004).

**Table 2:** Results of the forward regression analyses with the different fatigue scores described as dependent variables.

**Relapsing remitting MS patients**					
**DEPENDENT VARIABLE: COGNITIVE FATIGUE SCORE OF THE FSMC**					
**Model**	**Regression coefficient B**	***SD***	**Beta**	***T***	***p***
(Constant)	25.558	3.072		8.320	0.000
IL-1ß concentration	0.026	0.011	0.329	2.285	0.027
**DEPENDENT VARIABLE: MOTOR FATIGUE SCORE OF THE FSMC**					
(Constant)	17.941	3.226		5.561	0.000
Psychological BDI score	0.957	0.266	0.424	3.597	0.001
Disease modifying drugs	8.821	2.484	0.416	3.551	0.001
IL-1ß concentration	0.018	0.009	0.254	2.153	0.037
**DEPENDENT VARIABLE: FSS SCORE**					
(Constant)	31.825	9.598		3.316	0.002
Disease modifying drugs	11.939	4.354	0.334	2.742	0.009
IL-1ß concentration	0.051	0.015	0.415	3.398	0.002
Psychological BDI score	1.018	0.454	0.267	2.242	0.031
Age	−0.376	0.178	−0.263	−2.113	0.041
**Secondary progressive MS patients**					
**DEPENDENT VARIABLE: FSS SCORE**					
(Constant)	72.421	14.856		4.875	0.000
Age	−0.575	0.278	−0.383	−2.072	0.049
**Healthy controls**					
**DEPENDENT VARIABLE: COGNITIVE FATIGUE SCORE OF THE FSMC**					
(Constant)	13.500	1.162		11.637	0.000
Psychological BDI score	1.288	1.288	0.699	6.111	0.000
**DEPENDENT VARIABLE: MOTOR FATIGUE SCORE OF THE FSMC**					
(Constant)	14.548	1.250		11.642	0.000
Psychological BDI score	1.091	0.227	0.611	4.814	0.000
**DEPENDENT VARIABLE: FSS SCORE**					
(Constant)	28.387	4.415		6.430	0.000
Psychological BDI score	1.227	0.380	0.460	3.227	0.003
Age	−0.202	0.099	−0.291	−2.044	0.048

*BDI, beck depression inventory; FSMC, fatigue scale for motor and cognition; FSS, fatigue severity scale; MS, multiple sclerosis*.

## Discussion

The results of the present study demonstrated that MS patients with a secondary progressive disease course showed a somewhat increased level of IL-1ß in comparison to healthy controls and rrMS patients. Moreover, we found a significant effect of disease modifying treatment on the IL-1ß concentration. Patients receiving such a therapy showed a significantly lower IL-1ß concentration than patients not receiving such a therapy. Furthermore, the IL-1ß concentration was one of the main predictors of fatigue scores in rrMS patients. It did not predict fatigue scores of spMS patients and healthy controls.

As far as we know, a comparison of peripheral inflammation between rrMS and spMS patients has not been performed before. Many reasons may explain the higher IL-1ß level in the spMS group compared to rrMS patients and healthy controls, which are irrelevant for the topic of our investigation. To name just a few: as expected spMS patients showed a high EDSS score (about 6). This may lead to body immobility, less immune competence and consequently increased IL-1ß values (35). Also, the higher age of spMS patients might account for an increased level of systemic proinflammatory cytokines in this group (36). We also found that receiving a disease modifying therapy has a significant effect on the IL-1ß concentration. This finding stands in line with previous studies demonstrating a potential effect of a disease modifying therapy on cytokine production (20–23). The fact that less spMS patients than rrMS patients received a disease modifying therapy may also be relevant for the higher IL-1ß concentration in spMS patients as compared to rrMS patients and healthy controls. Irrespectively of the underlying causes for the increased IL-1ß concentration in spMS patients, the finding that spMS patients had a somewhat elevated concentration of IL-1ß might indicate that even in spMS patients there may be a link between inflammatory activity and the feeling of fatigue.

The findings of the regression analyses point to a relationship between the proinflammatory cytokine IL-1ß and fatigue only in MS patients suffering from a relapsing remitting disease course. The salivary concentration of IL-1ß was included in all models best predicting fatigue scores in rrMS patients. This finding stands in contrast to two previous studies that did not find a relation between fatigue and blood levels of IL-1ß in MS patients (16, 37). This difference might be due to the fact that Akali and colleagues did not use regression analyses to investigate the relation between fatigue and the IL-1ß concentration. They did not control for factors such as depression, MS type or disease modifying drugs (37). Also Malekzadeh et al. did not consider depressive symptoms in their statistical analysis (16). We did control for an effect of depressive symptoms on the IL-1ß concentration. Furthermore, we included a larger amount of MS patients than the two previous studies and we divided patients into rrMS and spMS patients and checked for a relationship between IL-1ß and fatigue in the separate groups. Our results suggest that especially in the early disease stages of MS, bodily inflammation may be one dominant cause for fatigue. However, the relatively low R^2^ scores indicate that IL-1ß alone may explain only a limited part of the variance in experienced fatigue. Other cytokines like TNF-α, IL-6, IL-12, or IL-17 may have an additional impact on subjective fatigue (7).

Overall, fatigue seems to be a multi-factorial symptom resulting from different causes. Besides immunological abnormalities, also structural brain changes may contribute to fatigue. Several studies found a relation between fatigue and gray matter atrophy within specific brain areas such as frontal motor areas and subcortical areas such as the thalamus and basal ganglia (38). Nevertheless, it is noteworthy that in rrMS patients IL-1ß best predicted fatigue scores, whereas in healthy controls the psychological items of the BDI appeared to best predict the variance of the different fatigue scores. Hence, the level of IL-1ß seems to play an important role in fatigue in rrMS patients, whereas—as shown in healthy controls—fatigue might also be related to depressive symptoms as argued in many prior investigations (39–41).

The absence of a significant difference in IL-1ß concentration between rrMS patients and healthy controls raises some questions. One may argue that the peripheral level of IL-1ß cannot explain subjective fatigue, because then rrMS patients should show a higher IL-1ß concentration than healthy controls. The lack of a significant difference in the IL-1ß concentration between rrMS patients and healthy controls may be due to the small group sizes. Furthermore, the majority of rrMS patients received a disease modifying therapy. We found that patients receiving such a therapy had a significantly lower IL-1ß concentration. Hence, the lack of a difference may also be due to fact that most rrMS patients received a disease modifying therapy. Nevertheless, several studies show that chronic neuroinflammation sensitizes the brain to produce an exaggerated response to peripheral inflammation resulting in prolonged sickness behavior and increased cytokine induction within the central nervous system (42, 43). MS is a chronic inflammatory disease of the central nervous system which is characterized by chronic peripheral and central inflammation. Hence, MS patients in general might be sensitized to the effects of peripheral proinflammatory cytokines. Consequently, MS patients might produce an exaggerated response to peripheral inflammation resulting in exaggerated fatigue, whereas peripheral inflammation has no effect on healthy controls. This might explain the finding that even though rrMS and healthy controls present a similar concentration of salivary IL-1ß, rrMS present significantly higher fatigue scores.

According to the regression analyses, age seems to have an influence on the Fatigue Severity Scale Score in healthy controls, rrMS and spMS patients. Age negatively correlated with the fatigue scores in all groups. This unexpected finding contradicts the assumption that increased age is related to higher fatigue scores.

Additional evidence for a relationship between fatigue and bodily inflammation in MS patients comes from recent studies in which we demonstrated the importance of inflammation-induced vagal nerve activity for the generation of fatigue. In a previous study (44), we found that fatigue severity predicts future relapses in MS patients, additionally pointing to a relation between bodily inflammation and fatigue severity in rrMS patients. We also showed that fatigue in MS patients is associated with afferent vagal nerve signaling. A disruption of afferent interoceptive signaling is related to the absence of fatigue in MS patients (45, 46). Furthermore, fatigue correlates with autonomic symptoms (47), especially with those that strongly depend on vagal nerve signaling such as bladder dysfunctions, orthostatic intolerance and pupillomotor dysfunctions.

Given that an elevated systemic concentration of IL-1ß causes fatigue in rrMS patients, anti-inflammatory interventions should have a positive effect on fatigue in this patient population. Recent studies did show that aerobic exercise and resistance training have a combined positive effect on proinflammatory cytokine concentration and on fatigue in mildly impaired MS patients (48–53). Moreover, substances with anti-inflammatory properties such as Alfacalcidol, vitamin A or coenzyme Q10 seem to have the potential to reduce subjective fatigue in MS patients (54–57). Other studies showed that blocking IL-1ß signaling via Anakinra, an IL-1ß receptor antagonist exerts a positive effect on fatigue in patients suffering from inflammatory diseases such as rheumatoid arthritis or Sjögren's syndrome (58, 59).

There is another finding of our study, not directly related to the question of IL-1ß level and fatigue, that should be mentioned. As expected, rrMS and spMS patients differed in age, disease duration and EDSS, but not in their total and cognitive fatigue level. Consequently, the argument that fatigue results from a loss of function and a compensatory effort to solve everyday problems is less likely. Only the motor fatigue score showed a significant difference between the groups. Motor fatigue is strongly associated to muscle strength and motor impairment and might reflect the impact of motor impairment rather than the actual feeling of fatigue (60, 61). If the compensatory effort model of fatigue is correct, then it will concern motor fatigue more than cognitive fatigue.

One limitation of this study is that salivary IL-1ß concentration may not only reflect systemic immune responses but also local immune responses. This might explain the high number of outliers for the salivary IL-1ß concentration scores. To exclude an extremely high IL-1ß concentration due to local inflammation in the mouth, oral examinations need to be included. Nevertheless, gingival crevicular fluid is not only the result of local cytokine production but it is also a fluid of systemic origin indicating systemic inflammation. Moreover, also other factors that were not assessed in this study such as obesity, smoking or stress may also increase bodily inflammation and might have influenced the high concentration of IL-1ß in spMS patients (62–64). Hence, we cannot draw conclusions on causality regarding the relation between fatigue and IL-1ß in MS patients. Future studies on inflammatory markers in MS patients should also check for a potential effect of the above mentioned factors on peripheral inflammation.

## Conclusion

We recently developed a model for MS-related fatigue arguing that the feeling of fatigue is caused by systemic inflammation, resulting from inflammation-induced activity changes within interoceptive brain areas (7). The present study points to an association between the salivary concentration of the proinflammatory cytokine IL-1ß and subjective fatigue in rrMS patients. We also found that a disease modifying therapy significantly lowered the IL-1ß concentration. Future studies combining immunological and radiological measures are needed for a better understanding of the relation between subjective fatigue scores, peripheral immune markers and structural and functional changes in the central nervous system.

## Author contributions

KH: elaboration of study design, data collection, saliva analysis, writing of manuscript. CS and LQ: data collection, saliva analysis. H-PS and AK: help on patient recruitment, facilitation of examination room and materials. MH: help on patient recruitment, data collection, facilitation of examination room and materials. PE: writing of manuscript. HH: elaboration of study design, writing of manuscript.

### Conflict of interest statement

The authors declare that the research was conducted in the absence of any commercial or financial relationships that could be construed as a potential conflict of interest.
